# Chronic Renal Failure and Cardiovascular Disease: A Comprehensive Appraisal

**DOI:** 10.3390/jcm11051335

**Published:** 2022-02-28

**Authors:** Keren Skalsky, Arthur Shiyovich, Tali Steinmetz, Ran Kornowski

**Affiliations:** 1 Department of Cardiology, Rabin Medical Center, Petah Tikva 4941492, Israel; arthur.shiyovich@gmail.com (A.S.); ran.kornowski@gmail.com (R.K.); 2 Sackler Faculty of Medicine, Tel Aviv University, Tel Aviv 6997801, Israel; noptabi@yahoo.com; 3 Department of Nephrology, Rabin Medical Center, Petah Tikva 4941492, Israel

**Keywords:** chronic kidney disease, coronary artery disease, coronary angiography

## Abstract

Coronary artery disease is highly prevalent in patients with chronic kidney disease. The concomitant renal disease often poses a major challenge in decision making as symptoms, cardiac biomarkers and noninvasive studies for evaluation of myocardial ischemia have different sensitivity and specificity thresholds in this specific population. Moreover, the effectiveness and safety of intervention and medical treatment in those patients is of great doubt as most clinical studies exclude patients with advance CKD. In the present paper, we discuss and review the literature in the diagnosis, treatment and prevention of CAD in the acute and chronic setting, in patients with CKD.

## 1. Introduction: The Scope of the Clinical Problem

Coronary artery disease (CAD) is highly prevalent in patients with chronic kidney disease (CKD) [1], with a linear increase in the risk of cardiovascular mortality with decreasing eGFR [2,3]. The latter is reported to be twice as high in patients’ estimated glomerular filtration rate (eGFR) of 30–59 mL/min/1.73 m^2^, compared to individuals with normal kidney function, and three times higher at eGFR of 15–29 mL/min/1.73 m^2^ [4]. Concomitant renal disease poses a major challenge in decision making as symptoms, cardiac biomarkers and noninvasive studies for evaluation of myocardial ischemia have different sensitivity and specificity thresholds in this specific population. Iodinated contrast agents should be used with extreme caution to prevent further deterioration of renal function secondary to contrast induced nephropathy (CIN). Moreover, the effectiveness of coronary intervention in those patients is of great doubt as most clinical studies exclude patients with advance CKD. The resulting consequence might be the undertreatment of cardiovascular risk factors and inappropriate, low rates of coronary angiography in patients with CKD, also known as “renalism” [5]. Our aim herein is to review the literature in the diagnosis and treatment of CAD, in the acute and chronic setting, in patients with CKD.

## 2. A Brief Review of the Pathophysiology

The pathophysiology of combined cardiovascular and kidney disease extends across several interfaces. First, conventional risk factors for atherosclerosis can affect both organs with coronary artery disease, renal artery stenosis, endothelial dysfunction and small vessel disease [6]. Second are the hemodynamic interactions, including resistant hypertension, fluid overload and major alterations in blood pressure with abnormal regulatory response [7]. Next is the activation of the renin–angiotensin–aldosterone system, which is recognized in both CKD and heart failure, and plays an important role in the maintenance of cardiovascular homeostasis [8,9]. Anemia and chronic inflammation can contribute to the overlap of morbidities, along with uremic toxins [10]. Lastly, the mineral-bone disorder complicating CKD causes hyperphosphatemia and positive calcium balance, which stimulate vascular calcifications, accelerated atherosclerosis and structural changes in the heart [11]. The phrase “cardiorenal syndrome” represents the mutual influence of the acute or chronic dysfunction of the heart or kidney on the other organ [12].

## 3. Basic Definitions

CKD is defined as the abnormality of kidney structure or function, present for more than 3 months, with implications for health. It is classified based on the eGFR category and albuminuria category. On the basis of eGFR and urine albumin measurements, chronic kidney disease is classified into six stages of eGFR (G1, 2, 3A, 3B, 4, and 5) and three proteinuria stages (A1, 2, and 3) [13]. In this review, we use the eGFR categories, detailed in Table 1.

## 4. Chronic Coronary Syndrome and Renal Failure

### 4.1. Noninvasive Diagnostic Tests

Among patients with CKD, there are limited options for non-invasive diagnostic tests. The recently published European Society of Cardiology (ESC) guidelines regarding chronic coronary syndrome suggest using either coronary CTA or functional stress imaging as the preferred methods for risk stratification [14]. In patients with CKD, the risk of contrast-induced kidney damage is increased [3], and thus the use of coronary CTA is less acceptable. Moreover, patients with CKD, and especially those under dialysis, tend to have a high calcium score, which can reduce the coronary CTA specificity [15].

The next optional non-invasive testing would be the myocardial perfusion single-photon emission computed tomography (SPECT) test and stress echocardiography. Since patients with CKD, and especially advanced CKD and dialysis, have limited exercise capacity due to muscle fatigue, peripheral vascular disease, peripheral neuropathy, anemia, volume overload and other comorbidities, the Bruce protocol is not always possible. The alternative would be a dobutamine stress echocardiography (DSE) or SPECT scan with dipyridamole/adenosine infusion.

DSE is considered a good prognostic tool for the predictive evaluation of patients with CKD [16], but among renal transplant candidates, its sensitivity and specificity for the diagnosis of CAD was found be as low as 52% and 74%, respectively [17]. A single center experience of CAD screening using DSE in 40 hemodialysis patients found 0 sensitivity of the test. Authors concluded that, in CKD patients, the decision regarding coronary angiography should be based on other noninvasive tests and cardiovascular risk factors [18]. Moreover, adverse effects during DSE testing are relatively frequent, precluding the achievement of a target heart rate in about 5 to 10% of tests [19].

A systematic review of the literature published by Wang et al. in 2011 examined the accuracy of non-invasive cardiac screening tests, compared with coronary angiography in kidney transplant candidates. The specificity and sensitivity of both DSE and SPECT scans were highly variable. Overall, 13 studies examining the accuracy of DSE had a pooled sensitivity of 0.79 (95% CI 0.67 to 0.88) and specificity of 0.89 (95% CI 0.81 to 0.94), and 9 studies examining the SPECT test performance had a pooled sensitivity of 0.74 (95% CI 0.54 to 0.87) and specificity of 0.70 (95% CI 0.51 to 0.84) [20].

Another prospective study of 138 kidney transplant candidates found that SPECT had a low sensitivity (53%) and modest specificity (82%) for detecting obstructive CAD (≥50% stenosis). Among the patients with a normal SPECT, 14% of the patients had obstructive CAD on invasive angiography [21]. Marwick et al. found that while the sensitivity of dipyridamole thallium scintigraphy for detecting obstructive CAD was 95% in the non-CKD control group, it was only 37% in dialysis patients even after matching for the severity and extent of CAD [22]. In this study, the probability of obstructive CAD in patients with normal SPECT was 38%. Finally, in the ISCHEMIA-CKD trial published recently, which included patients with advanced kidney disease (defined as an estimated glomerular filtration rate (eGFR) of <30 mg/dL), and moderate-to-severe ischemia on noninvasive testing, a total of 26.5% of the patients had no obstructive CAD despite the pathological noninvasive test [23].

A few explanations for the reduced sensitivity and specificity of the myocardial perfusion SPECT in CKD/ESRD patients were suggested: an abundance of multivessel disease resulting in balanced, global ischemia; the presence of collateral vessels that produce the appearance of more homogeneous perfusion and a false-negative SPECT; and higher resting levels of adenosine, resulting in a higher resting coronary flow and reduced responsiveness to dipyridamole, resulting in “inadequate functional stress” [22].

### 4.2. The Cardiac Catheterization, Coronary Revascularization Strategies and Pharmacotherapy

A retrospective study of patients on renal replacement therapy, aiming to determine the sensitivity and specificity of various noninvasive ischemic tests, found the thallium dipyridamole scintigraphy to have 80% sensitivity and only 37% specificity. The authors of the article concluded that “angiography seems to be the only method to clearly document CAD in patients on renal replacement therapy” [24]. Nevertheless, the evidence regarding the efficacy of cardiac catheterization in the non-acute setting in patients with CKD is conflicting, as most cardiovascular trials excluded patients with advanced kidney disease. Moreover, there is paucity of data to confirm the prognostic benefit of angiography or even coronary revascularization among stable coronary patients with CKD.

A small trial published in 1992 randomly assigned 26 kidney transplant candidates with diabetes and obstructive CAD to medical therapy versus revascularization. A combined cardiovascular endpoint occurred in 10 of 13 medically managed patients, compared to 2 of 13 revascularized patients, within 8.4 months [25]. The ISCHEMIA-CKD published in 2020 aimed to examine the efficacy of initial invasive strategy versus conservative management in patients with advanced CKD (GFR < 30 mg/dL) and suspected ischemia. The trial found no evidence that an initial invasive strategy can reduce the risk of death or nonfatal myocardial infarction. However, patients with severe anginal symptoms, heart failure or recent acute coronary syndromes (ACS), or ejection fraction of less than 35%, were excluded from the trial, and thus the results do not extend to those high-risk patients [23]. Moreover, only 50.2% of the patients in the invasive group underwent revascularization, and in the conservative-strategy group, the 3-year cumulative incidences of revascularization (e.g., “crossover strategy”) was 19.6%.

The use of an iodinated contrast agent should be addressed aiming at minimizing exposure, and every effort should be made to minimized the potential damage to the kidneys, as detailed in chapter B.2 [3,4,26].

Overall decisions regarding the diagnostic and treatment modalities in CKD patients with suspected chronic coronary syndrome, should be tailor-made with careful consideration of the patient’s complaints, medical history, laboratory tests, images, renal function and prognosis. As a part of the integrated and multifactorial approach, the “heart team” forum should consult with nephrologists, diabetologists and primary care physicians on how to optimize the medical care for the heart, the kidneys and the cardiovascular risk factors altogether. This approach has been recently proven to improve cardiovascular outcomes in diabetic patients with CKD in a multicenter randomized control trial, with a long durability of protection [27].

#### 4.2.1. Revascularization Options

Revascularization options in patients with CKD include coronary artery bypass grafting (CABG) surgery and percutaneous coronary intervention (PCI). A meta-analysis published in 2017, including 11 studies with 29,246 patients comparing PCI with drug eluting stents (DES) versus CABG in patients with CKD and multivessel disease. The study found that CABG was superior to PCI-DES in the long-term outcomes. That included all-cause mortality, myocardial infarction (MI), repeat revascularization and the composite outcome of major adverse cardiac and cerebrovascular events (MACCEs). The disadvantage of CABG over PCI was in the short-term mortality and stroke rates. Subgroup analysis restricted to patients on renal replacement therapy yielded similar results, but no significant differences were found regarding stroke and MACCE [28].

A post hoc analysis of the SYNTAX (The Synergy between Percutaneous Coronary Intervention with Taxus and Cardiac Surgery) trial on patients with CKD found similar results in favor of open heart surgery, with a significant advantage being given to CABG over PCI (MACCE rate of 31.5% vs. 42.1%, *p* = 0.019) in the five-year follow up. Results were driven by repeat revascularization and all-cause death. Differences were more pronounced among diabetic patients, and a similar trend, although non-significant, was demonstrated among non-diabetic patients [29]. The SYNTAX score principles for allocating patients to PCI or CABG are similar for patients with a normal or abnormal kidney function [30].

#### 4.2.2. The Dual Antiplatelet Therapy (DAPT)

DAPT with aspirin and an oral P2Y inhibitor is the mainstay of antithrombotic therapy after PCI. The ESC guidelines published in 2019 on the diagnosis and management of chronic coronary disease suggest four treatment options for combination therapy with aspirin in patients who have a high or moderate risk of ischemic events: clopidogrel, ticagrelor, prasugrel and low dose rivaroxaban [14]. Nevertheless, there is limited data to support new P2Y12 inhibitors, ticagrelor and prasugrel, over clopidogrel in patients with CKD after elective PCI.

The duration of DAPT therapy for stable CAD is 6 months. This may be shortened to 3 months in patients with a risk of high bleeding, or it may be prolonged to 12 months in those with a high risk of stent thrombosis and ischemic events [14]. Patients with advanced kidney disease (GFR < 30mg/dL) have both a high risk of ischemic events and high bleeding risk, as calculated by the HASBLED score, and thus pose a challenge to treatment [2,14,31,32,33]. A prespecified sub study of the TWILIGHT trial (The Ticagrelor with Aspirin or Alone in High-Risk Patients After Coronary Intervention) showed that, among CKD patients undergoing PCI, ticagrelor monotherapy for 9 months following 3 months of dual antiplatelet therapy with ticagrelor and aspirin, reduced the risk of bleeding without a significant increase in ischemic events as compared with ticagrelor plus aspirin for 12 months [34]. The balance between efficacy and bleeding should be considered for each patient individually when choosing the therapeutic regimen and the duration of the treatment.

### 4.3. Advanced CKD: Pre-Dialysis, Dialysis and Renal Transplant Candidates—Different Populations

The definition of advanced CKD has been generalized. Patients can be categorized into three subgroups: pre-dialysis patients, those on renal replacement therapy and renal transplant candidates. Each subgroup has unique features to be considered when choosing the diagnostic studies and treatment options in CAD.

Pre-dialytic patients should be carefully examined to prevent further deterioration of renal function, including avoiding certain drugs (as discussed in the next chapter) and minimizing iodine contrast exposure. The clinical presentation in this group may be conflicting as it is difficult to distinguish between effort dyspnea resulting from volume overload and that secondary to significant CAD stenosis. The consideration of nephrotoxic drugs and iodine contrast exposure are less crucial in patients already on renal replacement therapy, although there is even more limited data on the prognostic efficacy of drug therapy and PCI in this subgroup of patients, as the proportion of dialytic patients in clinical trials in mostly very small.

The third subgroup of patients is the renal transplant candidates. The prevalence of CAD in this subgroup is very high and reported to be 42–81% [35]. Positive non-invasive stress testing in this population is predictive of augmented death during follow-up [36], although there is no evidence that screening for CAD improves survival or reduces cardiac events [20]. A post hoc analysis of the ISCHEMIA CKD trial according to the kidney transplant list status was recently published. The study examined intervention versus conservative management in 194 participants listed for transplant, compared to 583 patients not listed. The results suggest that the overall prognosis of renal transplant candidates is better than the prognosis of those not on the list, but there was no evidence to support improved survival from preemptive coronary revascularization in patients on the waiting list. Although this sub-study was concluded as negative, it is important to note that the sample size was small and there was non-trivial cross over, with 22% of the patients randomized to the conservative strategy eventually underwent coronary revascularization. Moreover, only 51 patients underwent transplantation, and there is no data of the outcomes after renal transplantation in patients managed with conservative strategy or invasive strategy [37]. Treatment considerations in patients on the waiting list should include the potential future damage to the transplanted kidney with the need of performing coronary revascularization post-surgery. This might be especially relevant in living kidney transplant candidates, in which the timing of the coronary intervention and surgery can be planned in advance.

### 4.4. Prevention of Future Events

Medical treatment for risk-factor control (lipids, blood pressure and glucose) can improve outcomes.

#### 4.4.1. Blood Pressure (BP) Control

Hypertension (HTN) can be both the cause and the outcome of advance CKD. It is the second most important cause of CKD after diabetes, and is an independent risk factor for other cardiovascular events, including stroke, MI, heart failure and peripheral artery disease [38]. A large meta-analysis of 18 randomized controlled trials and 15,924 patients with CKD, have shown a significant reduction in all-cause mortality following intensive BP control [39].

The evidence about BP target in patients with CKD is inconsistent in clinical trials. Most studies suggest that BP should be lowered to <140/90 mmHg and towards 130/80 mmHg [40,41,42]. The recently published Kidney Disease Improving Global Outcomes (KDIGO) guidelines for the management of blood pressure further reduce the target systolic BP to <120 mmHg [43]. This is based on the SPRINT trial, a randomized control trial, that found the systolic BP target < 120 mmHg to reduce CV events and all-cause mortality, compared to a target of <140 mmHg [44]. To note, the SCORE classification used for cardiovascular risk assessment in hypertensive patients is not applicable in CKD, as those patients are already defined at high or very high risk due to their renal disease [14]. In dialysis patients, the timing of BP measurement is important, and the most reproducible method is considered to be ambulatory BP monitoring between dialysis treatments over a period of 1 to 2 weeks [45,46].

BP reduction can be achieved with lifestyle advice and BP lowering medications. Renin angiotensin (RAS) blockers are effective in reducing albuminuria compared to other antihypertensive agents [40]. They were also found to reduce mortality, MI, stroke and congestive heart failure among patients with left ventricular dysfunction, previous vascular disease and high-risk diabetes. Current guidelines recommend a combination of a RAS blocker with a CCB as the preferred initial anti-HTN therapy [47]. Additional treatment options include the addition beta-blockers, spironolactone (with extra caution in CKD) or another additional diuretic therapy (amiloride, thiazide or thiazide-like diuretics or loop diuretics), alpha-blockers, centrally acting agents (e.g., clonidine), or minoxidil [48].

Two other drugs have emerged in clinical practice recently, both originally prescribed for glycemic control, as detailed in the next section, but might have mild beneficial impact on BP control. Sodium glucose co-transporter 2 (SGLT2) inhibitors were found to lower BP by 7–10 mmHg [49,50,51], and glucagon-like peptide-1 receptor agonists (GLP-1RAs) have a more modest effect on BP, reducing it by 1–5 mmHg [52].

BP reduction by antihypertensive agents can lead to a mild increase in serum creatinine, but this does not necessarily reflects renal injury [53,54]. Nevertheless, each therapeutic agent should be considered and monitored carefully to assure its tolerability and impact on potassium level and renal function. Special attention should be given when using RAS blockers and spironolactone in patients with eGFR < 45 mL/min/1.73 m^2^ and serum potassium levels > 5.0 mmol/L.

#### 4.4.2. Glycemic Control

Diabetes mellitus (DM) is associated with about a two-fold excess risk for cardiovascular diseases, regardless of other conventional risk factors [55]. Only patients with type 1 DM aged under 35 years or type 2 DM aged under 50 years, with a short duration of the disease (less than 10 years) and no other risk factors, can be classified as moderate risk (1–5%) for a fatal cardiovascular event within 10 years. All other diabetic patients are classified at either high risk (5–10%) or very high risk (>10%) [31]. CKD in patients with DM is associated with higher rates of cardiovascular disease and should be considered in the highest risk group for prognostication and/or therapeutic management [55,56,57]. Special attention should be given to diabetic patients with abnormal proteinuria, as its presence further increases cardiovascular events and mortality [58]. Considering the high prevalence of diabetic patients developing CKD, the annual screening of kidney function and albumin secretion by blood and urinary testing is required in DM patients. About 30% of patients with type 1 DM and 40% with type 2 DM develop CKD.

Glycemic control was found to reduce microvascular and macrovascular complications on long-term follow up [59,60,61]. The target a near-normal HbA1c (<7.0% or <53 mmol/mol) was found to be effective in reducing microvascular complications, while evidence for an HbA1c target to reduce macrovascular risk is less persuasive.

The options for medical therapy to control diabetes varies according to renal function. Metformin can be used safely in patients with moderately reduced renal function (i.e., eGFR > 30 mL/min), and results in a lower risk of death and heart failure hospitalization compared with insulin and sulfonylureas [62,63]. As eGFR drops towards 30 mL/min, its use should be considered with cation as it might be associated with death in end-stage renal disease (ERSD). Metformin should be withheld before cardiac catheterization to prevent lactic acidosis, although a contemporary trial suggest that metformin continuation, compared with a 48 h interruption, was not associated with clinical lactic acidosis [64]. If acute kidney injury occurs post angiography, the regimen should be stopped for 48 h or until renal function has returned to its initial level [31].

Similarly, SGLT2I medications can also be used in the same renal function category (eGFR > 30 mL/min), and are beneficial in reducing glycosylated hemoglobin and improving glycemic control, with the additional benefit of reducing cardiovascular morbidity and mortality, heart failure hospitalizations and preventing further deterioration in kidney function in both diabetic and non-diabetic patients with CKD [49,50,51]. GLP1RAs can be used in patients with more advanced renal failure, up to eGFR of 15 mL/min, and has been shown to have nephroprotective characteristics and reduced urinary albumin excretion, as well as an additive weight reduction effect [65].

As kidney function deteriorates, treatment options are more limited, and the use of insulin instead of oral regimens is more common and safe. In dialysis patients, the reliance on the conventional measures of glycemic control, including hemoglobin A1C, is debatable. Dialysis patients experience alterations in glycemic control due to decreased kidney function, uremic milieu and dialysis therapies. A phenomenon known as “burnt-out diabetes” may occur as glycemic control improves spontaneously, leading to reduced glycated hemoglobin levels and necessitate the cessation of antidiabetic drugs. Moreover, the low level of HbA1C may be falsely lowered due to a short lifespan of red blood cells and increased proportion of young erythrocytes in the blood of dialysis patients [66,67]. However, there is evidence that both high (>8%) and low (<6%) levels of glycated hemoglobin are associated with poor outcomes in dialysis patients, and it is thus reasonable to recommend an A1c range of 6–8% as the desired target of treatment [68].

#### 4.4.3. Lipid Control

Patients with advanced CKD (eGFR < 30 mL/min) are considered to be at high (stage G3b CKD) or very high risk (stage G4 and G5 CKD or on dialysis) of cardiovascular disease, and there is no need to use risk estimation models in these patients [32]. According to ESC guidelines, the target LDL is 70 mg/dL or 55 mg/dL in high and very high risk patients accordingly [32]. However, the KDIGO consensus document suggest that CKD patients do not need routine follow-up measurements of lipid level, as the LDL cholesterol levels do not necessarily suggest the need to increase statin doses [69]. This may be partially explained by elevated triglyceride levels, lowered HDL-C levels and a shift of LDL subclasses to small dense LDL particles in concordance with the deteriorating kidney disease [70]. Moreover, in patients with advanced CKD, efficacy and safety issues of lipid-lowering agents should be carefully considered, prior to the initiation of treatment and dose escalation.

An individual patient data meta-analysis of 28 trials and ~180,000 CKD patients published in 2016, examined the effect of statin-based therapy on major vascular events according to the CKD level. Overall, statin-based therapy was found to reduce the risk of a first major vascular event by 21% per 1 mmol/L reduction in LDL cholesterol. Efficacy became smaller as eGFR declined, with only little evidence of benefits in patients on dialysis. Researchers concluded that in patients with chronic kidney disease, the goal is to achieve the largest possible absolute reduction in LDL-C to maximize the treatment benefits [71]. The KDIGO organization developed updated practice guidelines for the management of dyslipidemia in CKD patients. The use of statins or the statin/ezetimibe combination is recommended in non-dialysis patients with CKD stage G3-5. As for dialysis patients, in patients already on lipid-lowering agents at the time of dialysis initiation, the continuation of these drugs should be advocated, especially in the presence of atherosclerotic cardiovascular disease (ASCVD). In dialysis patients who are free of ASCVD, the initiation of statin is not recommended [69].

Most statins are metabolized mainly by the liver and cleared minimally by the kidney, thus dose adjustment with chronic kidney disease and hemodialysis is not necessary [72]. The hydrophilic statins pravastatin and rosuvastatin are exceptional as their renal excretion is more pronounced and pose an increased risk of myopathy and rhabdomyolysis in CKD patients [73]. The dose adjustment of rosuvastatin is indicated with non-dialysis severe renal impairment (CKD stage G3-5) [74]. The initial recommended dose is 5 mg once daily and the maximal recommended dose is 10 mg a day.

Special attention should be given to kidney transplant recipients, as the concurrent use of certain immunosuppressive agents may have adverse effects on lipid metabolism. On the one hand, it may cause lipid disturbances and, on the other hand, it may result in an increased risk of myopathy or rhabdomyolysis due to drug interactions. Evidence of increased myopathy exist for the combination of cyclosporin and statins metabolized by cytochrome P450 (CYP)3A4 (lovastatin, simvastatin, atorvastatin and pravastatin) and for fluvastatin metabolized by CYP2C9 [75]. Tacrolimus seems to have no major interaction with statins [76]. Interestingly, the use of atorvastatin has been proposed to improve kidney function after transplantation, presumably due to its anti-inflammatory and immunomodulatory properties [77]. Mild proteinuria is sometimes seen with a high-dose statin treatment, but current evidence suggest that this is not associated with a deterioration in renal function [73,78].

Monoclonal antibodies targeting proprotein convertase subtilisin/kexin 9 (PCSK9) reduce low-density lipoprotein cholesterol (LDL-C) and lipoprotein(a) in high-risk populations. In a sub-group analysis of CKD patients, evolocumab was found to be superior to placebo and ezetimibe when added to statins, and its clinical efficacy and safety were consistent across CKD groups [79]. In a similar analysis of the therapeutic regimen arilocumab, the treatment was found to be effective in patients with CKD compared to patients with normal renal function [57]. Both trials did report outcomes in patients with advanced kidney disease undergoing dialysis.

#### 4.4.4. Lifestyle Modifications

Implementing healthy lifestyle behaviors further decreases cardiovascular risks among patients with CKD. Lifestyle recommendations include smoking cessation, physical activity and maintaining a healthy weight, similar to individuals without kidney injury [80]. As for dietary recommendations, a low-protein diet mitigates proteinuria and helps to preserve renal function for a longer duration, which is recommended in the early stages of CKD. Sodium restriction is recommended to control fluid overload and hypertension and to improve the cardiovascular risk profile. As for potassium, restricted intake is only recommended in patients with advanced stages of kidney disease with a tendency toward hyperkalemia, along with a diet rich in fruits and vegetables. Restricted dietary phosphorus intake is widely recommended for patients with moderate-to-advanced kidney disease not receiving dialysis, even in the absence of apparent hyperphosphatemia. This is in order to prevent the secondary increase in fibroblast growth factor 23 (FGF-23) and parathyroid hormone, which can cause collateral renal, bone and heart damage [81,82].

Key massages for the diagnosis, treatment and prevention approach in patients with chronic coronary syndrome and CKD are summarized in Table 2.

## 5. Acute Coronary Syndrome and Renal Failure

### 5.1. Cardiac Biomarkers in the Presence of Renal Failure

Increased levels of high-sensitivity cardiac troponin (hs-cTn) are necessary for the diagnosis of non-ST elevation myocardial infarction (NSTEMI) in the general population, and are broadly used in clinical practice to distinguish unstable angina from NSTEMI [26]. Impaired renal function is one of the major confounders of cardiac troponin concentration [32]. Increased levels of the cardiac biomarker troponin in patients with CKD are common. The raised values typically reflect the continuous myocardial damage caused by long-term exposure to uremic toxins, left ventricular hypertrophy, CAD and heart failure [83,84]. This might explain the fact that patients with troponin concentrations above 99th percentile have a two-fold greater risk of subsequent myocardial infarction or cardiac death at 1 year, regardless of the diagnosis [85,86]. Another suggested mechanism is reduced renal clearance causing increasing levels of troponin over time, as renal function deteriorates [84,87]. Recently published clinical trial in patients presented to the hospital with suspected ACS found that elevated hs-cTn levels increased as kidney function declined, from 10% in patients with normal kidney function to 66% at an eGFR of less than 30 mL/min/1.73 m^2^. The proportion of patients with type 1 myocardial infarction decreased from 74% to 35% [88].

These data make the interpretation of the laboratory results and the diagnosis of NSTEMI more challenging. In CKD patients, the 0/1 h hs-cTn algorithm of the ESC was found to have comparable sensitivity of rule out (i.e., a threshold of <5 ng/L can be used to rule out myocardial injury). Nevertheless, the specificity of rule in and overall efficacy was decreased [89]. Furthermore, those patients might have ECG abnormalities associated with electrolyte abnormalities and left ventricular hypertrophy, which make clinical evaluation even more challenging. Therefore, ECG changes should be differentiated from old abnormalities and absolute changes in cardiac troponin should be assessed when considering the diagnosis of acute MI [26].

### 5.2. The Cardiac Catheterization, Coronary Revascularization Strategies and Pharmacotherapy

Although individuals with CKD have a worse prognosis in the setting of MI than individuals with normal renal function, they are less likely to receive an early invasive strategy and potent P2Y12 inhibitors as recommended by the guidelines [90,91,92,93]. Data from the SWEDEHEART registry published in 2009 found that an early invasive strategy in patients with NSTEMI and CKD stage G2 to G4 is associated with better outcomes and greater 1-year survival. The benefit declined with lower renal function, and is less certain in those with stage G5 CKD or on dialysis [94]. A more recent study on both STEMI and NSTEMI patients found invasive management to be associated with significantly lower in-hospital mortality in comparison to a conservative approach in all CKD stages, including patients on hemodialysis [95].

When choosing an invasive strategy, measures should be taken to prevent acute chronic kidney injury and CIN, as those may increase the risk of major adverse cardiovascular events and mortality. The definition of acute kidney injury is based on an elevation of ≥0.3 mg/dL in creatinine levels 48 h post PCI [96], although recent publications suggest using serum and urine biomarkers for the early diagnosis of the complication, before the expected serum creatinine increases. Suggested biomarkers vary and include Cystatin C, neutrophil gelatinase-associated lipocalin (NGAL), β2-microglobulin and inflammatory cytokines IL18 and TNFα [97,98,99]. Current ESC guidelines recommend the use of low volume iso or hypo-osmolar contrast materials during the cardiac catheterization of individuals with CKD. Adequate hydration prior and post intervention is the mainstay of acute kidney injury prevention, with the administration of 1 mL/kg/h isotonic saline 12 h before contrast exposure, and continued for 24 h after the procedure. To prevent over-congestion, the recommended volume of fluids is lowered to 0.5 mL/ kg/h if left ventricle ejection fraction is lower than 35% or New York Heart Association (NYHA) functional classification is above 2 [3,26,30]. High-dose statins were also described as beneficial [100].

The principles leading to the decision about the revascularization method are similar to those detailed in chapter A.2. CABG should be considered over PCI in suitable patients with multivessel CAD, whose surgical risk profile is acceptable and life expectancy is above 1 year [101,102].

The choice of antiplatelet agents should be considered carefully according to each individual bleeding and ischemic risk as CKD is one of the risk factors for both bleeding and ischemic events. Nevertheless, in the clinical scenario of ACS, the dual antiplatelet therapy was found to have significant net benefits in preventing cardiovascular events among patients with renal insufficiency [26]. Specifically, the treatment with ticagrelor in patients with CKD and ACS compared with clopidogrel was found to reduce ischemic events and mortality with no significant increase in major bleedings. Data from the PLATO (Platelet Inhibition and Patient Outcomes) trial suggested that the absolute reduction of ischemic events in CKD patients treated with ticagrelor was even more pronounced compared to those with normal renal function (4.7%/year vs. 1%/year) irrespective to the therapeutic strategy (conservative vs. invasive; percutaneous vs. surgical). The trial excluded patients receiving dialysis [103]. The efficacy and safety of prasugrel compared to ticagrelor were tested in the ISAR-REACT 5 trial (Intracoronary Stenting and Antithrombotic Regimen: Rapid Early Action for Coronary Treatment). A post hoc analysis of the trial according to eGFR categorized patients into three groups: low eGFR (<60 mL/min/1.73 m^2^), intermediate eGFR (≥60 and <90 mL/min/1.73 m^2^), and high eGFR (≥90 mL/min/1.73 m^2^). Prasugrel was found to be associated with lower occurrence of the primary outcome (a composite of all-cause death, myocardial infarction, and stroke) compared to ticagrelor in all eGFR categories, whereas bleeding rates were comparable for both drugs [93]. Dialysis patients were excluded from the trial. Finally, in CKD patients with additional high-risk features who have tolerated DAPT, it is reasonable consider long duration with ticagrelor 60 mg twice daily on top of aspirin beyond the initial year of treatment [104].

Key massages for the diagnosis and treatment in patients with acute coronary syndrome and CKD are summarized in Table 3.

#### Gaps of Knowledge and Future Trends

Evidence on the diagnostic and therapeutic management of CAD in patients with CKD is limited. The existed trials often consider patients with renal insufficiency of any degree as a homogenous group, which is not necessarily correct. Moreover, patients with more advanced kidney failure (stage G4 CKD and hemodialysis) are often excluded from pivotal trials and thus there is a lack of data upon their optimal cardiac management. The efficacy and safety of each therapeutic regimen and method discussed in the review should be tested in designated trials of patients with CKD, categorized according to their CKD level, including patients undergoing dialysis therapy.

## Figures and Tables

**Table 1:** GFR categories in CKD.

GFR Category	GFR (mL/min/1.73 m^2^)	Terms
G1	≥90	Normal or high
G2	60–89	Mildly decreased
G3a	45–59	Mildly to moderately decreased
G3b	30–44	Moderately to severely decreased
G4	15–30	Severely decreased
G5 *	<15	Kidney failure

* 5D and 5T indicate end-stage renal disease patients who undergo chronic dialysis (5D) treatment or have undergone kidney transplantation (5T).

**Table 2:** Key messages in chronic coronary syndrome and kidney disease.

Diagnosis	DSE and SPECT tests may be both used for the diagnosis of coronary artery disease, although their sensitivity and specificity is relatively low.
Treatment	Evidence regarding the efficacy of cardiac catheterization in the non-acute setting is conflicting. Efforts should be made to minimize the potential damage of iodinated contrast agent to the kidneys. CABG is superior to PCI in long-term outcomes. The SYNTAX score principles for allocating patients to PCI or CABG is similar to patients with normal or abnormal kidney function.
Advanced CKD (3 different populations)	Pre-dialysis—Patients should be carefully examined to prevent further deterioration of renal function, including avoiding certain drugs and minimizing iodine contrast exposure. Dialysis—The consideration of nephrotoxic drugs and iodine contrast exposure are negligible. Kidney transplant candidates—The risk assessment should include the potential future damage to the transplanted kidney with the need of coronary revascularization post-surgery.
Prevention of future events	BP should be lowered to <140/90 mmHg and towards systolic BP of <120 mmHg. In CKD stage G1–4, the target of near-normal HbA1c (<7.0%) is recommended. In hemodialysis patients, HbA1c range of 6–8% is preferred. Patients with advanced CKD are considered to be at high or very high risk of cardiovascular disease and there is no need to use risk estimation models. The use of statins or statin/ezetimibe combination is recommended in non-dialysis patients with a target LDL of 55 mg/dl. The use of statins among dialysis patients is debatable.

**Table 3:** Key messages in acute coronary syndrome and kidney disease.

Diagnosis	Increased levels of the cardiac biomarker troponin are common, thus absolute changes in cardiac troponin should be assessed when considering the diagnosis of acute MI.
Treatment	Early invasive strategy in ACS and CKD stage G2 to G4 is preferred. CABG should be considered over PCI in suitable patients with multivessel CAD, whose surgical risk profile is acceptable and life expectancy is above 1 year. The treatment with new P2Y12 inhibitors, ticagrelor and prasugrel, in patients with CKD and ACS, is preferred over clopidogrel.

## Data Availability

All authors take responsibility for all aspects of the reliability of the data presented.
